# Morbidity and Mortality of Statutory Versus Private Health Insurance Patients in Cardiac Surgery in Germany and a Critical Appraisal of the Current Health Insurance Situation

**DOI:** 10.3390/healthcare14203475

**Published:** 2026-10-16

**Authors:** Purevsuren Tsegmid, Filip Schröter, Viyan Sido, Julia Hübner, Martin Hartrumpf, Johannes M. Albes

**Affiliations:** Department of Cardiac and Vascular Surgery, Heart Center Brandenburg, University Hospital Brandenburg Medical School, 16321 Bernau bei Berlin, Germany; purevsuren.tsegmid@mhb-fontane.de (P.T.); viyan.sido@gmx.de (V.S.); julia.huebner@immanuelalbertinen.de (J.H.); martin.hartrumpf@immanuelalbertinen.de (M.H.); johannes.albes@mhb-fontane.de (J.M.A.)

**Keywords:** statutory health insurance, private health insurance, outcome, cardiac surgery, early mortality

## Abstract

**Background:** In all, 90% of patients in Germany are insured with statutory (SHI) instead of private health insurance (PHI). PHI offers significantly better benefits including earlier and more comprehensive diagnosis and therapy, which can reduce the complication rate and even mortality in fields where patients with high morbidity are treated, yet data on potential outcome differences between SHI and PHI patients in Germany is scarce. **Methods:** Between January 2023 and December 2025, 144 PHI and 2094 SHI patients underwent heart surgery at one institution. Preoperative baseline characteristics, risk factors, and peri- and postoperative information, including the surgeon’s experience level and distance of the referring hospital, were retrospectively compared. In addition, a propensity score matching (PSM) analysis was performed. **Results:** The SHI group presented with significantly higher BMI and EuroSCORE II, fewer male patients and higher NYHA states. Significantly more PHI patients were operated on by very senior staff members and none by a trainee, and their hospitalization time was significantly shorter. Neither early mortality (PHI 3.47%, SHI 5.35%, *p* = 0.432) nor distance levels of referring hospitals differed significantly. After PSM, only differences in the hospitalization time and surgical experience level remained significant. Early mortality came down to 4.86% in the SHI patients after PSM. **Conclusions:** In a multimorbid patient group, we found no evidence for a difference in outcome depending on insurance status, aside from a shorter hospital stay for PHI patients. Nonetheless, the situation in the pre- and post-hospital phases for SHI patients may have room for improvement. Efforts should be made to give all patients the same rapid and comprehensive access to healthcare services, increasing the system satisfaction of patients with SHI. In cardiac surgery, fortunately, there is no particular need for adjustments regarding the in-hospital phase.

## 1. Introduction

In Germany, statutory health insurance (SHI) and private health insurance (PHI) companies exist side by side. After the founding of the Federal Republic of Germany in 1949, the existing professional health insurance funds were given legal security and could continue to operate on the one hand but could also be newly established on the other. This has led to the current existence of 94 statutory health insurance funds, which provide insurance coverage for compulsorily insured persons but are also open to voluntarily insured persons who, due to their higher income, have the choice between statutory and private health insurance. Over the past 22 years, mergers have steadily reduced the number from over 1000 to the current level (Figure 1). Further mergers are planned in order to reduce administrative costs.

Private health insurance can be supplementary, but it can also replace statutory insurance entirely. In contrast to statutory health insurance, which is financed by contributions, private health insurance is funded by capital and is therefore, assuming sound capital management, more resistant to demographic and economic influences than statutory health insurance. In Germany, it is considered the insurance of choice for “high earners.” There are currently 33 private health insurance companies offering comprehensive insurance. Insured persons pay significantly lower premiums in their younger years than those insured under statutory health insurance, but the contribution progression is steeper than that in statutory health insurance, so that contributions are higher in old age than they are in statutory health insurance. Market share varies greatly. Leaving aside future mergers, which could suddenly change market shares, Debeka Krankenversicherung is currently the market leader (Figure 2).

The range and scope of benefits offered by private health insurance are significantly better than those offered by statutory health insurance. The gap between private and statutory health insurance is steadily widening, as benefits that were previously paid for by statutory health insurance are being cut in order to get spiraling costs under control (Table 1).

However, due to demographic developments, both systems, like all other social security systems (e.g., unemployment, pensions, long-term care in old age), are now under financial pressure and forced to steadily increase the premiums paid by insured persons, although the curve is steeper in SHI than in PHI [3] (Figure 3).

The actual benefits provided by PHI in the event of a claim are subject to the individual contractual arrangements but are generally more comprehensive and of higher value than those in SHI. In addition, PHI pays registered doctors and hospitals better so that their patients are more lucrative. Due to the shortage of specialists in Germany, this has also led to PHI patients being given preferential treatment over SHI patients and having faster access to the healthcare system, meaning they receive diagnosis and treatment earlier when they fall ill, despite all political efforts to ensure equal treatment. SHI patients now often wait weeks for an appointment with a general practitioner or months for an appointment with a specialist [4,5]. Some practices are even no longer accepting new SHI patients because of budget ceilings, which do not exist for PHI patients. In hospitals, admission appointments are also allocated much more restrictively to SHI patients than to PHI patients (Figure 4).

In Germany, around 90% of patients are insured with SHI and 10% with PHI. This raises the question of whether the low-threshold access of PHI patients to the healthcare system leads to better care than for SHI patients. In fields where patients with high morbidity are treated, earlier and more comprehensive diagnosis and therapy can reduce the complication rate and even mortality, on both outpatient and inpatient bases. In a study on patients undergoing colorectal surgery, survival did not differ between SHI and PHI patients, but it was shown that PHI patients more often received minimally invasive surgery [6]. These findings were later proven by a subsequent study by the same principal author. Additionally, it was found that lower income and living in rural areas also led to lesser use of minimally invasive approaches [7].

We wondered whether these results—the absence of significant differences in survival but presence of differences in treatment—also applies to patients who need to undergo heart surgery and retrospectively compared the complication rates and early mortality of all SHI and PHI patients at a heart center who were operated on and treated over a period of three years. Thus far, there is no data on potential outcome differences between SHI and PHI cardiac surgery patients in Germany. There is only one general register maintained by the German Society for Thoracic, Cardiac and Vascular Surgery, which records all patients regardless of their actual insurance status and provides an annual report on the results [8].

## 2. Materials and Methods

Between January 2023 and December 2025, 2238 patients underwent heart surgery in the Heart Center Brandenburg, which is a cardiac care center in a rural area north of Berlin, servicing a patient clientele of mixed rural and urban residency. Of these, 144 were insured by PHI and 2094 by SHI. All patients with a complete dataset from the clinical database were analyzed. Patient informed consent was waived due to the purely retrospective nature of the study and the complete anonymization. A respective ethics vote was obtained by the ethics committee of the Medical University the hospital belongs to (420042026-BO-E-RETRO, 21 May 2026). Preoperative baseline characteristics, as well as risk factors (e.g., EuroSCORE II [9]); the percentage of infectious endocarditis; the type of surgery; the surgeon’s experience level (trainee, junior board-certified surgeon, experienced staff surgeon, senior surgeon, deputy or head of department); postoperative intensive care stay; complications such as post-operative bleeding, re-thoracotomy, renal failure, pneumonia, sepsis, wound healing disorders, or stroke; length of stay in the intensive care unit; length of hospitalization; early mortality (defined as 30-day mortality); and the distance of the referring hospital were compared.

Statistical analysis was performed using R 4.6.0 (R Core Team, Vienna, Austria). In addition, a propensity score matching (PSM) analysis was performed with the R package MatchIt [10] using BMI, age, sex, ESII and urgency status as matching parameters and the nearest-neighbor matching method. Male patients were matched to female patients in a 1:1 ratio without replacement. A balance table and a Love plot were created using the cobalt package [11]. PSM selects patients from the control group with comparable preoperative baseline conditions to those in the treatment group, matching them as closely as possible to reduce bias before comparisons.

Numerical data is presented as means with standard deviations and was tested for normal distribution using the Shapiro–Wilk test before being compared by Student’s *t*-test or the Mann–Whitney U test. Categorical data was compared by Fisher’s exact test or the Chi^2^ test and is presented as total values and percentages. Ordinal data like NYHA state, the surgeon’s experience and the referring hospital distance was compared using the Cochran Armitage test for trends in proportions.

A power calculation was performed for the sample sizes before and after matching on the basis of a significance level of 0.05, a power of 0.8 and a two-sided hypothesis using the pwr package [12] in R. It revealed that before matching, the presented patient collective was large enough to detect a small-sized effect in the categorical variables (Cohen’s h = 0.241) and a small- to medium-sized effect for the numerical variables (Cohen’s d = 0.241). After matching, the sample size should still be enough to detect small- to medium-sized differences (Cohen’s h = 0.330, Cohen’s d = 0.331).

## 3. Results

### 3.1. Comparisons of Raw Data

BMI and EuroSCORE II were significantly higher in the SHI group compared to the PHI cohort, while the prevalence of male patients was lower (SHI 77.0%, PHI 87.5%, *p* = 0.005). Hospitalization time was significantly longer in the SHI than in the PHI cohort (Table 2, Figure 5).

There was a significant trend towards higher NYHA states in SHI patients, caused by a higher percentage of patients in clinical state NYHA I and a lower percentage in NYHA state III in the PHI cohort (Figure 6).

There was a significant (*p* < 0.001) trend towards a higher level of surgical experience amongst surgeons of PHI patients, driven by an almost twice-as-high percentage of patients being operated on by very senior staff members, as compared to SHI patients (Table 3). No PHI patients and only very few patients in the SHI cohort (1.62%) were operated on by residents in training under the supervision of experienced, board-certified cardiac surgeons. None of these patients died (Figure 7, Table 3).

The distance levels of the referring hospitals did not differ significantly between the two cohorts (Figure 8). This variable refers to the distance between the hospital that first admitted the patient and the Heart Center Brandenburg they were then referred to for treatment.

Neither early mortality (*p* = 0.432) nor other adverse postoperative incidents differed significantly between the PHI (3.47%) and SHI (5.35%) groups (Table 4).

### 3.2. Comparisons After Propensity Score Matching

Twenty patients were dropped due to incomplete datasets before PPM, which resulted in a balanced (see Supplementary S1 and S2) matched dataset of 144 patients per group. The overlap was also checked to be sufficient (Supplementary S3). Hospitalization time remained significantly different between the two groups, with 13.9 days in the PHI cohort versus 15.7 days in the SHI patients. Furthermore, the trend towards higher surgical experience level in surgeons of PHI patients remained significant (Figure 9). While the PHT level differed significantly between the two groups, this result is somewhat devalued by the high number of patients with unknown PHT status. All other parameters did not show any significant differences, just as before PPM. The early mortality rate was 4.86% in the SHI patients after PSM (Table 5, Table 6, Table 7 and Table 8).

## 4. Discussion

After homogenization of the baseline conditions such as age, sex, BMI, EuroSCORE II, and urgency status, privately insured patients did not show any significant directly outcome-related differences when compared to statutorily insured patients, regardless of the better benefits and more comprehensive diagnosis and care that PHI provides, which could have caused such differences. Due to the small sample size of PHI patients, this absence of evidence for a difference between the two groups cannot yet be seen as proof to exclude one. It might just be too small to detect on this level, thus raising the necessity for further research with a larger cohort. It should be noted, however, that the SHI patients experienced no significant disadvantages from these distribution differences. PHI patients tended to arrive in better overall condition at the hospital, with more patients being in NYHA state I as compared to the SHI patients and being slightly but significantly less obese than the latter ones. Both results could suggest that PHI patients are more aware of health-related issues than SHI patients, enabling them to make positive lifestyle adjustments and prompting them to make timely appointments with healthcare professionals [13]. This could be explored further by a study including questionnaires on health awareness in both groups. Another reason might be that insurance status is closely tied to socioeconomic status in Germany, which is likely an uncontrolled confounder in our study. However, this does not translate into faster access in emergencies, as both types of patients were admitted as emergency or urgent cases just as often. Their distribution of surgeons was roughly the same as that for patients with compulsory insurance, but PHI patients were operated on more often by highly experienced surgeons than SHI patients were. This is likely caused by optional insurance services that include the performance of the operation by a doctor chosen by the patient and are not covered by SHI but often are by PHI. In most cases, this is the head of the department or, in their absence, their permanent representative, who is usually the deputy surgeon in the department. This means that these patients were operated on by the doctors with the most experience who were therefore naturally best able to ensure the quality of the procedure, especially in the case of more extensive procedures. The optional service may also include the right to a single room or a two-bed room in the normal ward. This has no direct medical advantage, but it increases well-being and improves sleep at night because, for example, there are no restless or snoring fellow patients in the room and there are fewer nightly checks by the nursing staff. Thus, a single room can have a positive influence on the postoperative course. However, the phases of intensive care and the stay in the IMC, which contributed to roughly more than half of the entire length of stay, were excluded from this, so this factor can be classified as secondary. The hospitalization time for privately insured patients was shorter than that for patients with statutory health insurance. This may indeed be due, at least in part, to the single room situation, which leads to a faster recovery once the patient has reached the normal ward. In addition, there is no mandatory minimum length of stay for private patients. In contrast, this mandatory minimum length of stay should not be undercut for SHI patients, as otherwise the health insurance companies will apply deductions to the reimbursement. Since this system does not exist in the PHI system, these patients can be discharged without any budgetary restrictions based solely on their clinical condition. While significant, the reported difference in hospital stay seems to be too small to significantly influence outcomes. In summary, optional services can be considered advantageous for the outcome and the overall length of hospital stay for at least one third of PHI patients, but this is not statistically tangible.

We did not have robust data on the prehospital phase of the patients, so, aside from anecdotical reports by the patients, we were unable to statistically prove faster access to healthcare services and the associated acceleration and improvement in diagnosis and treatment for privately insured patients compared to compulsorily insured patients. Nevertheless, the trend toward a slightly facilitated course can at least be hypothetically linked to this given the current situation in Germany (Figure 4). In the analysis of the overall data before the propensity score analysis, we expected that privately insured patients would be much more likely to be elective or urgent cases than publicly insured patients and much less likely to be acute cases. Acute clinical situations can arise, among other things, as a result of delays in diagnosis and treatment. In this respect, this difference may indicate better pre-hospital care with faster referral to specialists and hospitals. Fully developed diseases with subsequent complications of other organs may therefore be less common. It can also be postulated that higher-income patients, who are naturally more likely to be privately insured, also have a higher level of education and, as a result, are more aware of impending illnesses, seek medical attention earlier, and are then able to get appointments faster [13,14]. This, however, was not evident in our patient collective. From a social perspective, such imbalance would indeed be disturbing [15]. It leads to envy among those with compulsory insurance towards patients insured directly by private health insurance and also towards those who are voluntarily insured in the statutory health insurance system with additional benefits corresponding to those offered by private health insurance, because the patients can afford the extra premiums. In cardiac surgery, however, all SHI patients can obviously be guaranteed that they will not experience disadvantages over PHI patients aside from a longer stay, which is due to the requirements placed upon the hospitals by the statutory health insurance companies rather than the actual clinical conditions in which they are submitted. Our study therefore confirms the findings in other fields that there are no significant differences in mortality and other negative outcomes that can be attributed to the type of health insurance.

Over the past 25 years, various federal governments have attempted to slow down, mitigate, or eliminate the explosion in healthcare costs and the increasing shortage of nursing and medical staff. In 2003, the former cost recovery principle was replaced by a performance-based flat-rate payment system for inpatient hospital treatment, the diagnosis-related groups (DRG) system, which was imported from Australia. Performance-based payment initially led to a stabilization of costs in favor of health insurance companies, but this was subsequently and gradually undermined by demographic developments and constant medical innovation, which led to steeply rising costs for medical care.

The last federal government publicly discussed merging statutory health insurance (translated into German: “GKV”) and private health insurance (“PKV”) into a so-called “Bürgerversicherung” (translated into English: “Citizens’ Insurance”) in order to provide equal care for all people in Germany as part of the desired concept of an egalitarian social system. However, this was not pursued further at first, as it turned out that private health insurance is relatively stable for at least 10% of citizens and only indirectly consumes tax money, as it is financed by capital. Furthermore, the transfer of private health insurance companies into the state system would have faced considerable legal hurdles.

After the COVID-19 pandemic, all problems in the healthcare system have ultimately worsened, including underfunding, staff shortages, increases in energy and material costs in hospitals, waiting times for outpatient appointments and hospital admissions. Somewhat helplessly, a law (Krankenhausversorgungs-Verbesserungsgesetz; KHVVG) has been introduced that has been amended by a subsequent adjustment law (Krankenhaus-Anpassungsgesetz; KHAG) due to its lack of enforceability. However, this is also unenforceable and has been returned to the legislature for re-examination [16]. In order to improve the situation for patients with statutory health insurance, it is imperative to improve access to outpatient and inpatient services in line with the situation for privately insured patients. In times of tight budgets, however, this can only be achieved by expanding outpatient care and optimizing the allocation of inpatient services. Politicians must stop merely scratching the surface of the existing system, and, in particular, they should not stir up envy between patients in the two systems. In the long term, the currently underfunded and ailing system, which covers 90% of the German population, should be reformed along the lines of the other system, which only provides health insurance for 10% of the population—not the other way around. Ideological barriers will not help.

A social security system that is at least partially funded and, thus, demographically secure is necessary for this and should be implemented. Ideally, this should be a two-pronged approach with state funding and early contributions to private capital stock. Individual contributions can be paid in equal parts by the employee and the employer, as is already the case with social security contributions, although in recent years an additional contribution beyond the 50:50 share, the actual amount of which is determined by the respective statutory health insurance company, called “Kassenindividuelle Zusatzleistung” (individual health insurance fund additional contribution), was passed initially on to the employee alone in order to reduce the burden on employers so as not to hinder industrial growth, as well as to maintain healthcare provision and avoid a growing deficit in statutory health insurance. This additional contribution was later converted into a contribution divided equally between the employee and the employer in order to avoid placing an ever-increasing burden on the employee alone. However, this additional contribution is rising steadily and will amount to a mean of 2.5% in 2025, up from 1.3% in 2021 [17]. Politicians have neglected this issue over the past 25 years and repeatedly postponed such a restructuring towards demographic security. In difficult economic times such as these, this affects 90% of the population, and the remaining 10%, who are currently relatively secure under the protection of the real money that secures their insurance, will ultimately also be caught up in this maelstrom.

### Limitations

This was a purely retrospective, single-center study involving a limited and recent patient population. Since only a minority of around 10% of patients in Germany are insured through PHI, their share of the study population (144) was lower compared to that of the SHI patients (2094), as expected. By including all patient data available in the reported time frame and using propensity score-matched analysis, we aimed at maximizing the study power and reliability of our reported outcomes. As mentioned in the Section 2, power analysis implied that the sample sizes should still be large enough to detect at least medium-sized differences between the populations.

The presented work is furthermore not comparable to a case–control study, as instead of comparing groups with known differences in outcome to find a possible cause for these, we compared two groups differing in a potential potent outcome predictor (extent and quality of medical treatment and care) by removing confounders using propensity score matching and examining whether their outcomes differed.

## 5. Conclusions

In the all-comer cohort we studied, patients with public health insurance had the same rapid and comprehensive access to medical care as did privately insured patients in the inpatient setting. How this perceived imbalance can be addressed politically and socially is the subject of heated debate across the political spectrum. It is clear, however, that substantial financial reserves are needed to offset demographic change and ensure intergenerational equity with regard to health and long-term care costs. Fortunately, in the day-to-day routine of cardiac surgery, there is no particular cause for concern regarding a noticeable imbalance. However, the periods before and after hospitalization may need improvement. This was not examined here and requires further study.

## Figures and Tables

**Figure 1:**
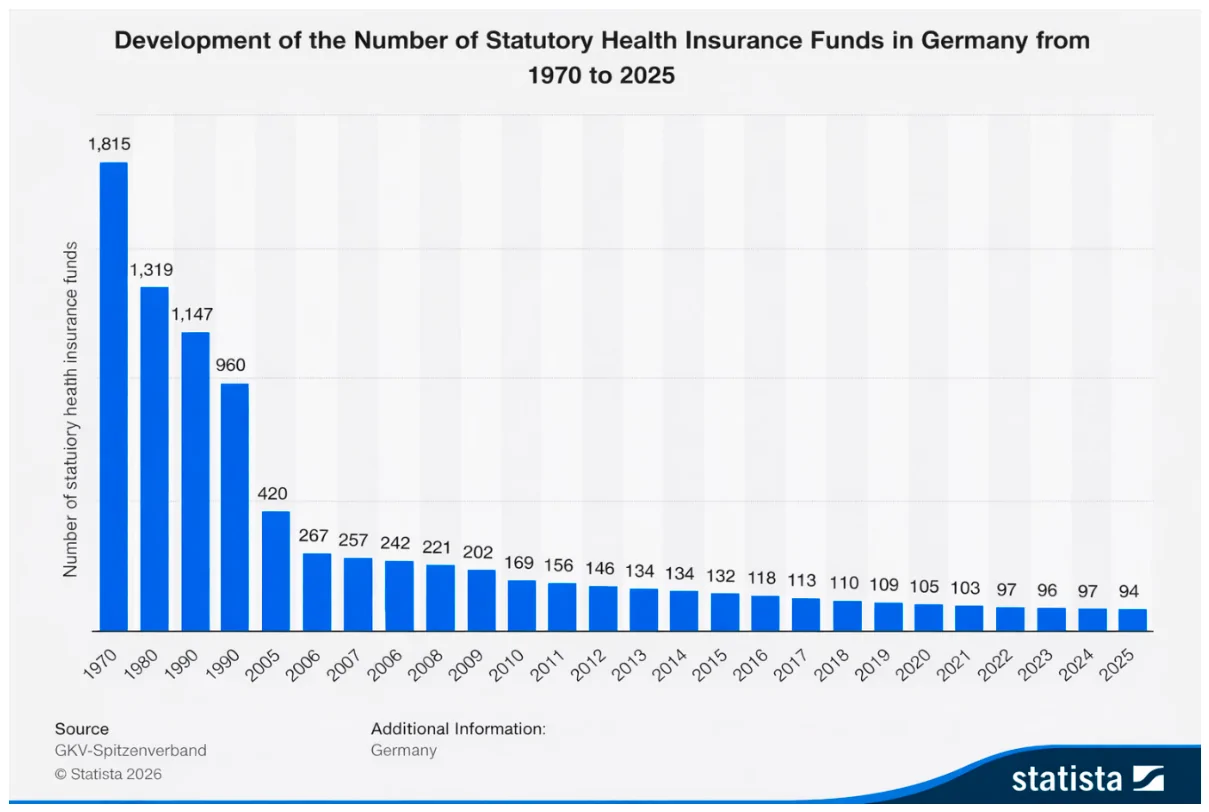
Decreasing number of statutory health insurance funds.

**Figure 2:**
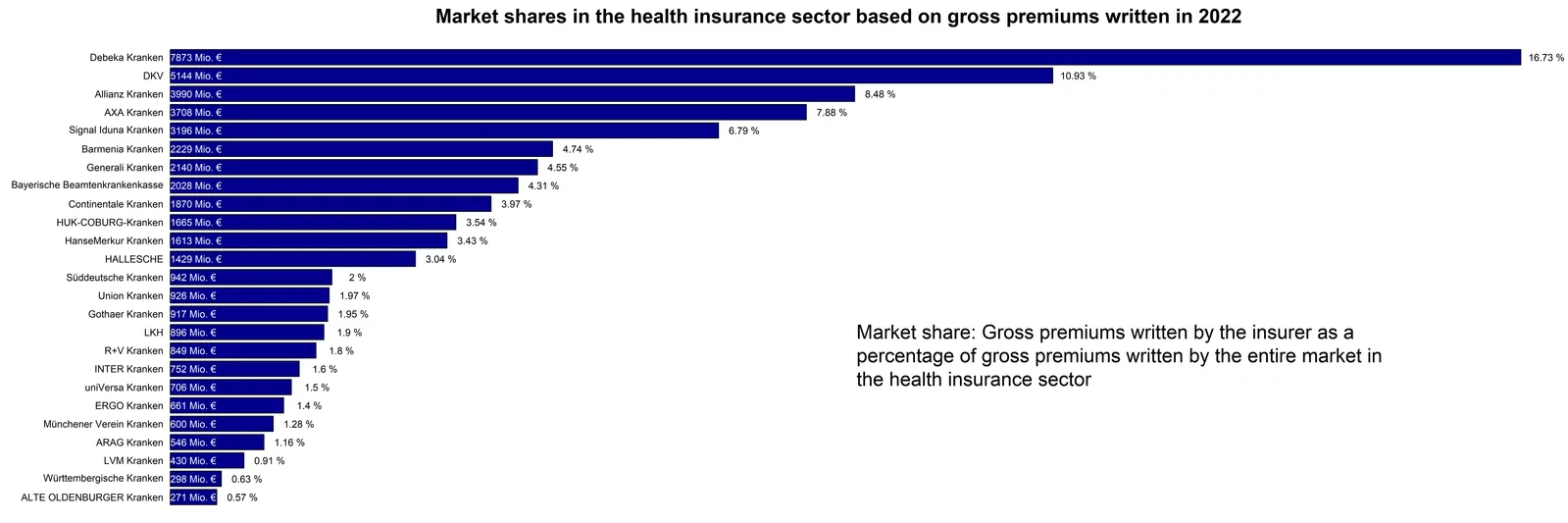
Market share of the 25 currently most valuable private health insurance companies in Germany [1].

**Figure 3:**
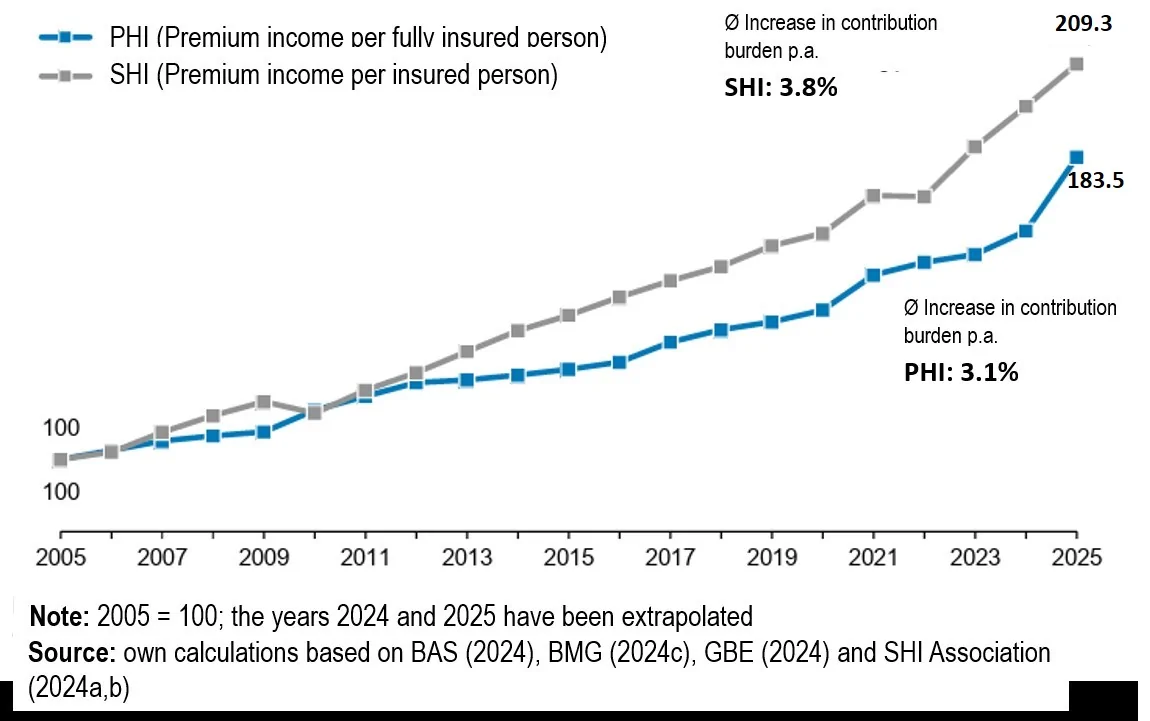
The development of contributions/premiums in SHI and PHI over the last 10 years [3].

**Figure 4:**
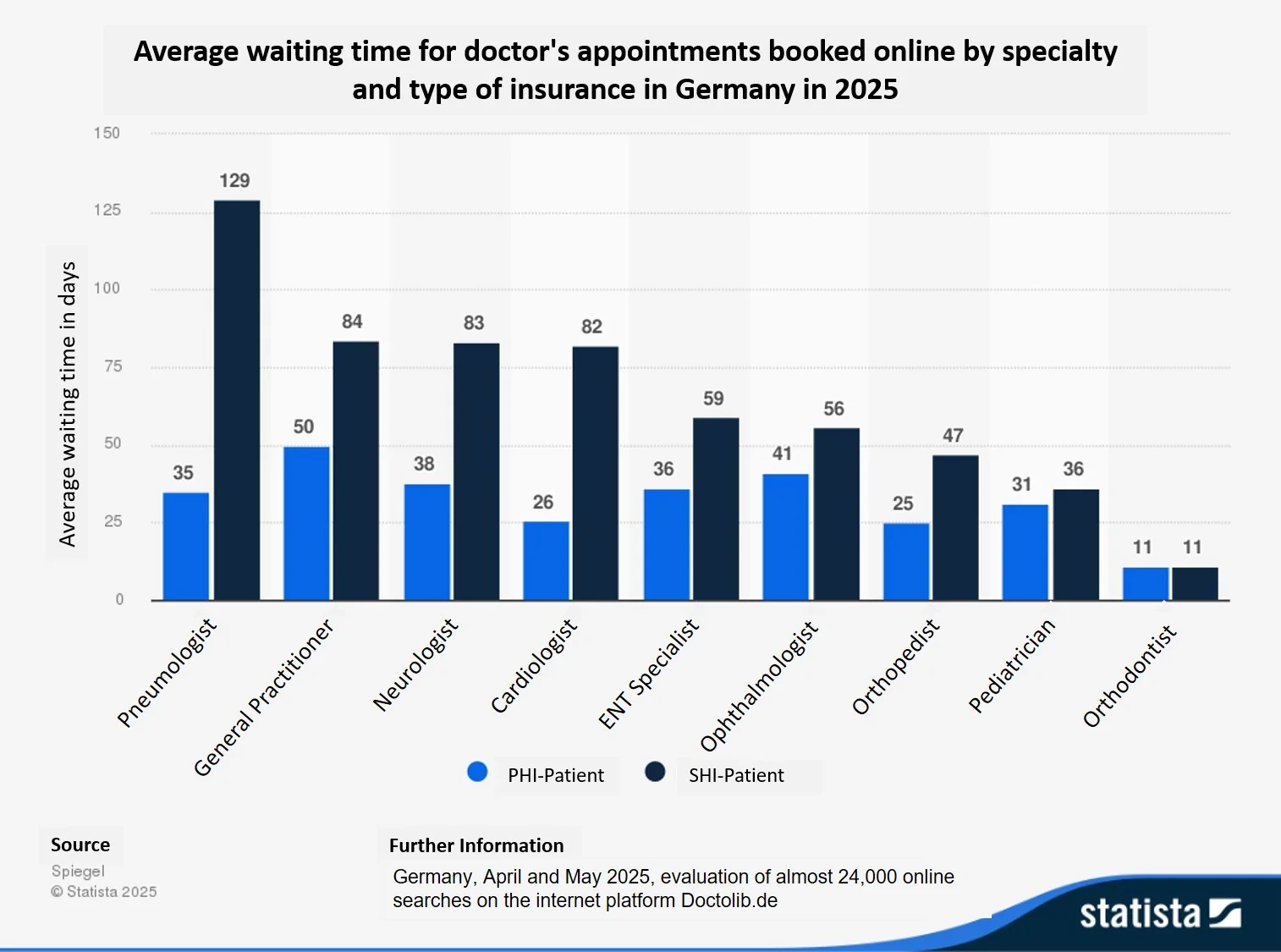
Waiting time for specialist outpatient appointment—difference between SHI and PHI.

**Figure 5:**
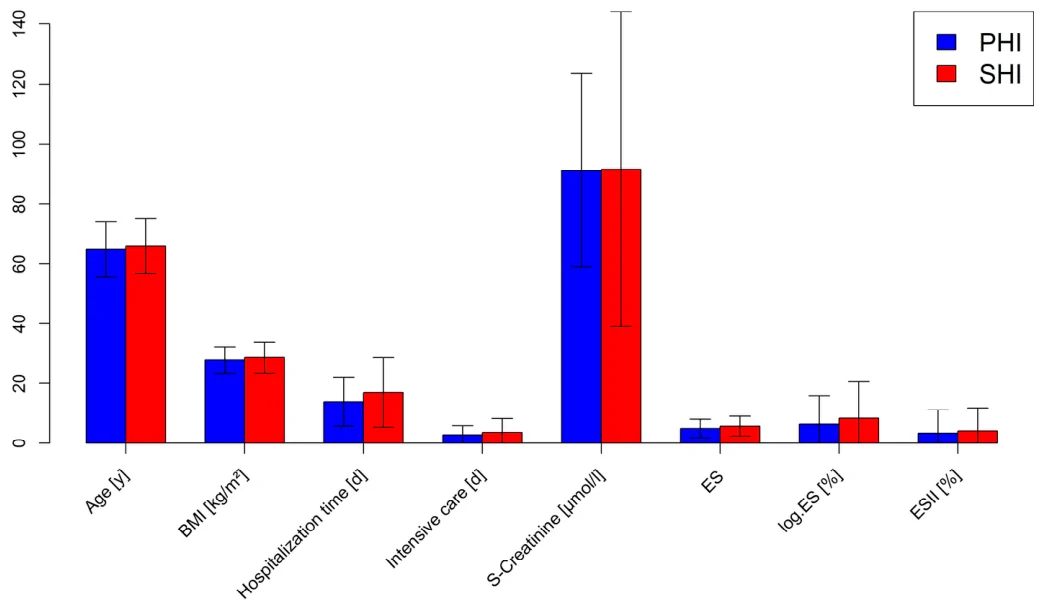
Baseline characteristics, mean ± SD.

**Figure 6:**
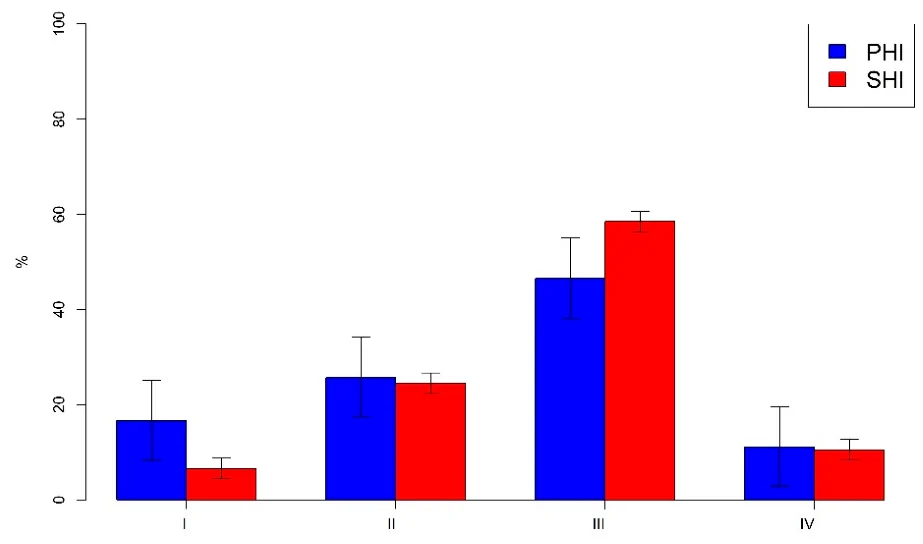
Preoperative NYHA state.

**Figure 7:**
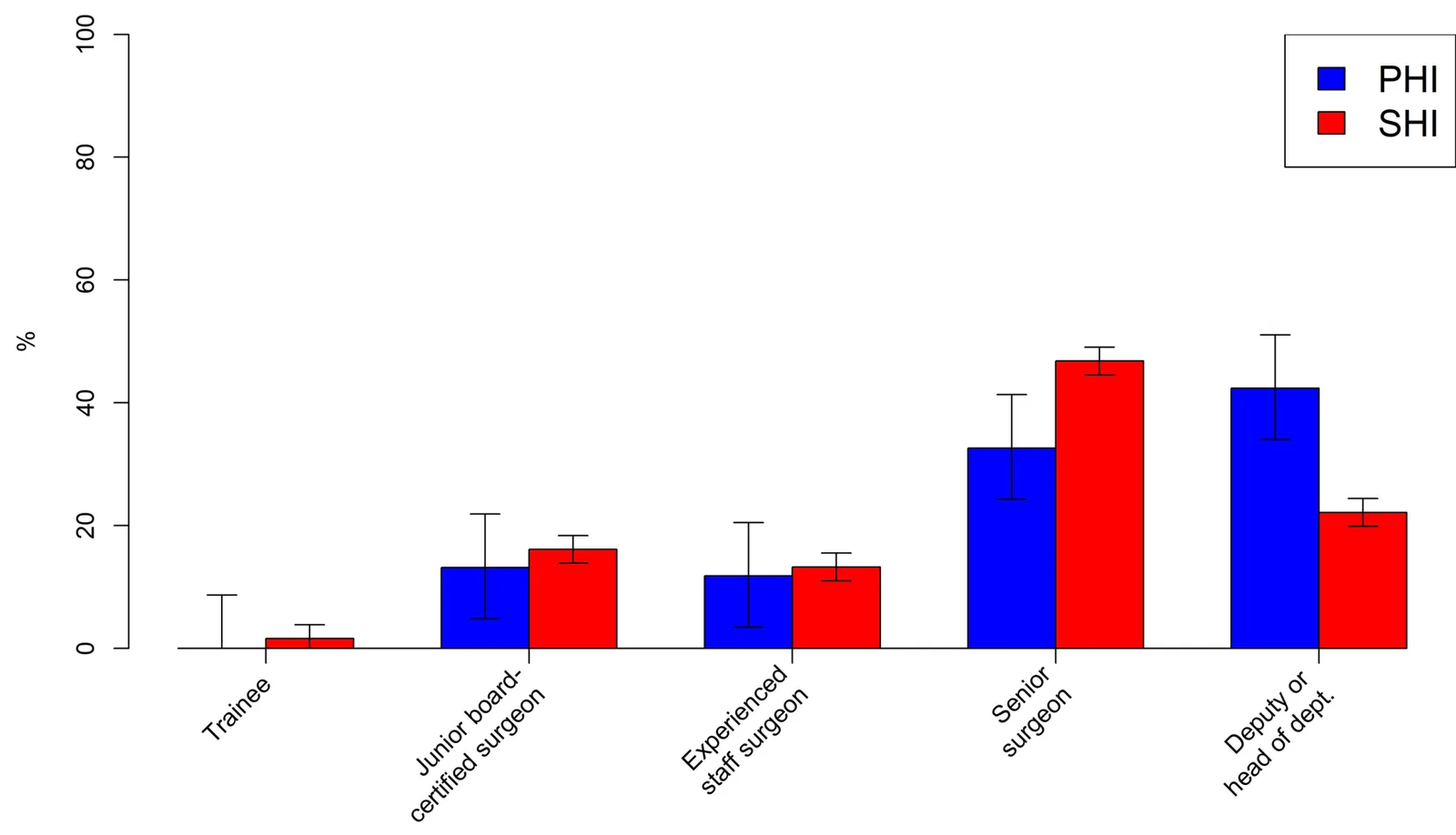
Surgeon experience level.

**Figure 8:**
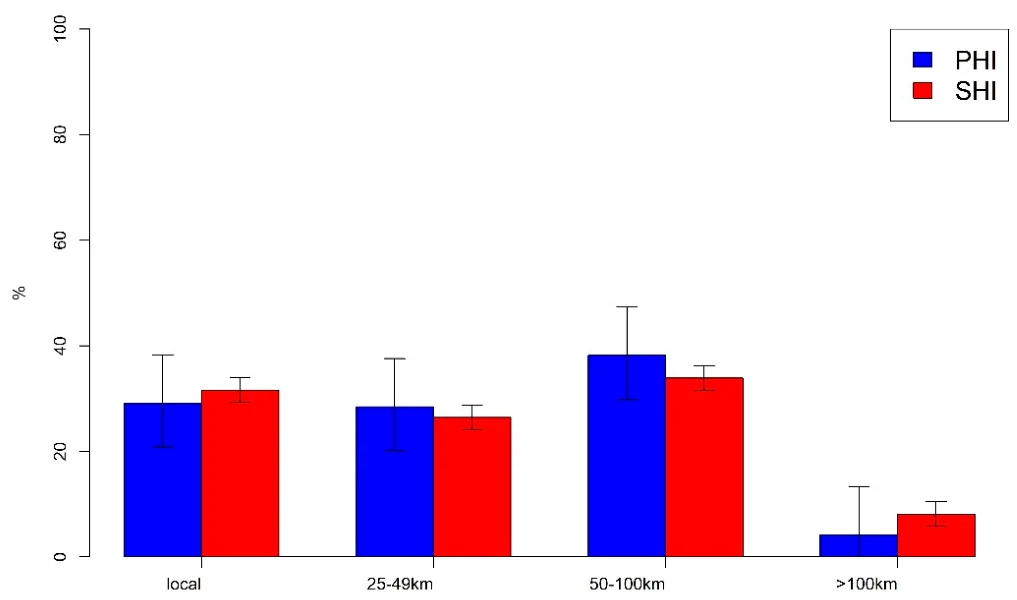
Referring hospital distance.

**Figure 9:**
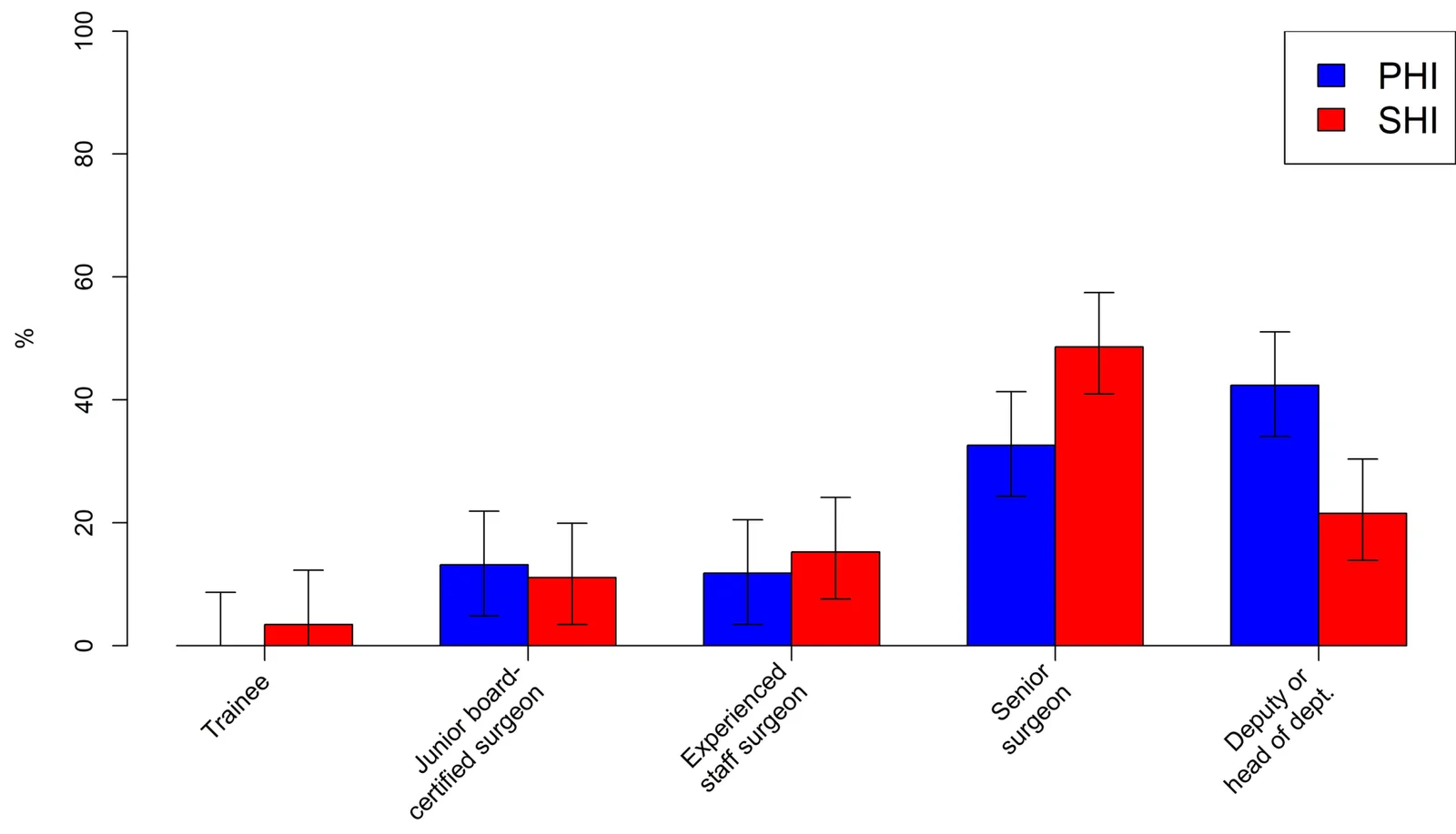
Surgeon experience level after propensity score matching.

**Table 1:** Performance profiles of PHI and SHI [2].

PHI	SHI
Free choice among all outpatient physicians	Care provided by panel doctors
Status as a private patient (e.g., faster appointments, short waiting times)	Referral required to see a specialist
All approved medications	Non-prescription medications only in exceptional cases
Reimbursement in the amount of actual costs	No coverage for minor health issues
	Rebate contracts determine reimbursable medications
	Reimbursement usually limited to fixed amounts
Free choice of hospital	Doctor determines hospital through referral
Often chief physician treatment	No entitlement to treatment by specific doctor
Often single- or double-occupancy room	Multi-bed rooms

**Table 2:** Unmatched patient data (baseline). Significant differences highlighted.

	PHI [144]		SHI [2094]		
	Mean	SD	Mean	SD	*p*
Age (y)	64.95	9.24	65.99	9.24	0.221
BMI (kg/m^2^)	27.83	4.44	28.67	5.18	**0.026**
Hospitalization time (d)	13.9	8.22	16.96	11.62	**<0.001**
Intensive care (d)	2.81	3.05	3.62	4.63	0.113
S-Creatinine (µmol/L)	91.29	32.26	91.66	52.61	0.179
ES	4.94	3.04	5.71	3.31	**0.004**
log. ES (%)	6.38	9.45	8.35	12.16	**0.003**
ES II (%)	3.4	7.69	4.12	7.67	**0.002**

**Table 3:** Unmatched patient data (organizational parameters). Significant differences highlighted.

	PHI [144]		SHI [144]		
	n	%	n	%	*p*
Surgeon experience (level)					**<0.001**
Trainee	0	0	34	1.62	
Junior board-certified surgeon	19	13.19	338	16.14	
Experienced staff surgeon	17	11.81	278	13.28	
Senior surgeon	47	32.64	980	46.8	
Deputy or head of dept.	61	42.36	464	22.16	
Referring hospital (distance level)					0.901
Local	42	29.17	662	31.63	
25–49 km	41	28.47	553	26.42	
50–100 km	55	38.19	709	33.87	
>100 km	6	4.17	169	8.07	

**Table 4:** Unmatched patient data (postoperative parameters).

	PHI [144]		SHI [144]			
	n	%	n	%	OR	*p*
OPCAB	5	3.50	78	3.74	0.93 [0.37–2.34]	1
Postop. Bleeding	0	0	8	0.38	0.85 [0.05–14.79]	1
Postop. Renal Failure	0	0	40	1.91	0.18 [0.01–2.87]	0.108
Emergency Re-Do	0	0	11	0.53	0.63 [0.04–10.69]	1
Postop. Infection	0	0	1	0.05	4.83 [0.2–119.08]	1
Postop. Sternum Instability	0	0	8	0.38	0.85 [0.05–14.79]	1
Postop. Hematoma	0	0	15	0.72	0.46 [0.03–7.8]	0.618
Surgical Access Complication	0	0	24	1.15	0.29 [0.02–4.83]	0.399
Postop. Mediastinitis	0	0	0	0	14.49 [0.29–733.2]	1
Postop. Resuscitation	0	0	22	1.05	0.32 [0.02–5.28]	0.394
Postop. Myocardial Infarction	0	0	5	0.24	1.31 [0.07–23.89]	1
Postop. Myocardial Ischemia	0	0	0	0	14.49 [0.29–733.2]	1
Postop. Neurological Dysfunction						0.846
None	144	100	2074	99.04		
TIA	0	0	4	0.19		
Stroke Minor	0	0	1	0.05		
Stroke Medium	0	0	7	0.33		
Stroke Severe	0	0	8	0.38		
Early Mortality	5	3.47	112	5.35	0.64 [0.26–1.58]	0.432

**Table 5:** Propensity score-matched patient data (baseline). Significant differences highlighted.

	PHI [144]		SHI [144]		
	Mean	SD	Mean	SD	*p*
Age (y)	64.95	9.24	63.85	8.55	0.106
BMI (kg/m^2^)	27.83	4.44	27.99	4.46	0.481
Hospitalization time (d)	13.9	8.22	15.69	9.04	**0.009**
Intensive care (d)	2.81	3.05	3.51	4.19	0.273
S-Creatinine (µmol/L)	91.29	32.26	88.47	25.37	0.567
ES	4.94	3.04	5.11	3.07	0.548
log. ES (%)	6.38	9.45	6.88	9.19	0.378
ES II (%)	3.4	7.69	3.22	3.9	0.125

**Table 6:** Propensity score-matched patient data (preoperative parameters).

	PHI [144]		SHI [144]			
	n	%	n	%	OR	*p*
Male	126	87.5	127	88.19	0.94 [0.46–1.9]	1
Active Endocarditis	9	6.25	13	9.03	0.67 [0.28–1.62]	0.5057
COPD	14	9.72	19	13.19	0.71 [0.34–1.47]	0.4593
PAOD	15	10.42	14	9.72	1.08 [0.5–2.33]	1
Chronic Dialysis	0	0	0	0	1 [0.02–50.74]	1
Critical Preop. State	5	3.47	4	2.78	1.26 [0.33–4.79]	1
STEMI Preop.	33	22.92	29	20.14	1.18 [0.67–2.07]	0.6671
Re-Do	3	2.08	4	2.78	0.74 [0.16–3.39]	1
Preop. Neurological Dysfunction						0.702
None	89	87.25	83	86.46		
TIA	9	8.82	10	10.42		
Stroke Minor	3	2.94	1	1.04		
Stroke Medium	1	0.98	2	2.08		
Stroke Severe	0	0.00	0	0.00		
IDDM						0.594
Unknown	72	50	73	50.69		
No DDM	32	22.22	32	22.22		
Diet	7	4.86	3	2.08		
NIDDM	19	13.19	25	17.36		
IDDM	14	9.72	11	7.64		
NYHA						0.025
I	24	16.67	8	5.56		
II	37	25.69	36	25		
III	67	46.53	86	59.72		
IV	16	11.11	14	9.72		
EF Preop.						0.606
Unknown	7	4.86	7	4.86		
Normal >50	103	71.53	90	62.5		
Moderate (31–50%)	25	17.36	44	30.56		
Poor (21–30%)	7	4.86	3	2.08		
Very Poor (<20%)	2	1.39	0	0		
PHT						**0.034**
Unknown	134	93.06	118	81.94		
None	6	4.17	6	4.17		
Minor (30–55 mmHg)	4	2.78	19	13.19		
Severe >55 mmHg)	0	0	1	0.69		
Urgency Status						0.927
Elective	81	56.25	78	54.17		
Urgent	53	36.81	56	38.89		
Emergent	8	5.56	10	6.94		
Ultima Ratio	2	1.39	0	0		

**Table 7:** Propensity score-matched patient data (postoperative parameters).

	PHI [144]		SHI [144]			
	n	%	n	%	OR	*p*
OPCAB	5	3.5	5	3.47	1.01 [0.29–3.56]	1
Postop. Bleeding	0	0	0	0	1 [0.02–50.74]	1
Postop. Renal Failure	0	0	2	1.39	0.2 [0.01–4.14]	0.498
Emergency Re-Do	0	0	1	0.69	0.33 [0.01–8.19]	1
Postop. Infection	0	0	0	0	1 [0.02–50.74]	1
Postop. Sternum Instability	0	0	0	0	1 [0.02–50.74]	1
Postop. Hematoma	0	0	3	2.08	0.14 [0.01–2.73]	0.247
Surgical Access Complication	0	0	3	2.08	0.14 [0.01–2.73]	0.247
Postop. Mediastinitis	0	0	0	0	1 [0.02–50.74]	1
Postop. Resuscitation	0	0	2	1.39	0.2 [0.01–4.14]	0.498
Postop. Myocardial Infarction	0	0	0	0	1 [0.02–50.74]	1
Postop. Myocardial Ischemia	0	0	0	0	1 [0.02–50.74]	1
Postop. Neurological Dysfunction						1
None	144	100	144	100		
TIA						
Stroke Minor						
Stroke Medium						
Stroke Severe						
Early Mortality	5	3.47	7	4.86	0.7 [0.22–2.27]	0.768

**Table 8:** Propensity score-matched patient data (organizational parameters). Significant differences highlighted.

	PHI [144]		SHI [144]		
	n	%	n	%	*p*
Surgeon experience (level)					**0.013**
Trainee	0	0	5	3.47	
Junior board-certified surgeon	19	13.19	16	11.11	
Experienced staff surgeon	17	11.81	22	15.28	
Senior surgeon	47	32.64	70	48.61	
Deputy or head of dept.	61	42.36	31	21.53	
Referring hospital (distance level)					0.612
Local	42	29.17	45	31.47	
25–49 km	41	28.47	45	31.47	
50–100 km	55	38.19	44	30.77	
>100 km	6	4.17	9	6.29	

## Data Availability

The individual-level data underlying this article is not publicly available because combinations of clinical characteristics may create a residual risk of re-identification. A data-minimized dataset may be made available to qualified researchers upon reasonable request, subject to institutional review, verification of the proposed research purpose and execution of an appropriate data-use agreement. No directly identifying information or re-identification key will be disclosed.

## Supplementary Materials

The following supporting information can be downloaded at https://www.mdpi.com/article/10.3390/healthcare14203475/s1, S1: Balance table; S2: Love plot; S3: Overlap histogram.

## Abbreviations

The following abbreviations are used in this manuscript:

PHI	Private health insurance
SHI	Statutory health insurance
NYHA	New York Heart Association
PSM	Propensity score matching
BMI	Body mass index
ES	EuroSCORE
log.ES	Logistic EuroSCORE
ES II	EuroSCORE II
SD	Standard deviation
OPCAB	Off-pump coronary artery bypass
Preop.	Preoperative
Postop.	Postoperative
TIA	Transient ischemic attack
COPD	chronic obstructive lung disease
PAOD	Peripheral arterial occlusive disease
STEMI	ST-elevation myocardial infarction
PHT	Pulmonary hypertension
IDDM	Insulin-dependent diabetes mellitus
NIDDM	Non-insulin-dependent diabetes mellitus
No DDM	No diabetes mellitus
EF	Ejection fraction
DRG	Diagnosis-related groups
KHVVG	Krankenhausversorgungs-Verbesserungsgesetz
KHAG	Krankenhaus-Anpassungsgesetz
